# Dorsal striatial hypoconnectivity predicts antipsychotic medication treatment response in first‐episode psychosis and unmedicated patients with schizophrenia

**DOI:** 10.1002/brb3.2625

**Published:** 2022-10-13

**Authors:** Eric A. Nelson, Nina V. Kraguljac, Jose O. Maximo, William Armstrong, Adrienne C. Lahti

**Affiliations:** ^1^ Department of Psychiatry and Behavioral Neurobiology University of Alabama at Birmingham Birmingham Alabama USA

**Keywords:** caudate, connectivity, first episode, psychosis, putamen, resting state, schizophrenia, striatum, treatment response

## Abstract

**Introduction:**

The dorsal striatum, comprised of the caudate and putamen, is implicated in the pathophysiology of psychosis spectrum disorders. Given the high concentration of dopamine receptors in the striatum, striatal dopamine imbalance is a likely cause in cortico‐striatal dysconnectivity. There is great interest in understanding the relationship between striatal abnormalities in psychosis and antipsychotic treatment response, but few studies have considered differential involvement of the caudate and putamen. This study's goals were twofold. First, identify patterns of dorsal striatal dysconnectivity for the caudate and putamen separately in patients with a psychosis spectrum disorder; second, determine if these dysconnectivity patterns were predictive of treatment response.

**Methods:**

Using resting state functional connectivity, we evaluated dorsal striatal connectivity using separate bilateral caudate and putamen seed regions in two cohorts of subjects: a cohort of 71 medication‐naïve first episode psychosis patients and a cohort of 42 unmedicated patients with schizophrenia (along with matched controls). Patient and control connectivity maps were contrasted for each cohort. After receiving 6 weeks of risperidone treatment, patients’ clinical response was calculated. We used regression analyses to determine the relationship between baseline dysconnectivity and treatment response.

**Results:**

This dysconnectivity was also predictive of treatment response in both cohorts.

**Discussion:**

These findings suggest that the caudate may be more of a driving factor than the putamen in early cortico‐striatal dysconnectivity.

## Graphical abstract

This study shows the presence of dysconnectivity between the dorsal striatum and largescale brain networks in psychosis spectrum patients, where reduced connectivity appears most prominent between the caudate and the default mode network as well as the putamen and areas of the salience network (most robustly seen in first episode psychosis patients). Baseline dysconnectivity of both striatal subregions was also predictive of subsequent antipsychotic treatment response, where greater baseline connectivity abnormalities were associated with poorer outcomes.

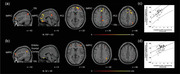

## 1 INTRODUCTION

The dorsal striatum is the largest subcortical region in the mammalian brain. It integrates multiple cortical and subcortical neuronal signals and is a critical structure for motor learning, voluntary movements, and decision‐making through convergence of sensorimotor, cognitive, and motivational information (Guo et al., 2015). The dorsal striatum is under predominant control of the dopamine signal, receiving direct dopamine projections via the nigrostriatal pathway (Abi‐Dargham & Moore, 2003; Gerfen et al., 1990; Matsuda et al., 2009), subsequently projecting to prefrontal and motor cortices (Herrero et al., 2002). While it is a singular structure in rodents, in humans, it is comprised of two distinctly separate nuclei, the caudate, and putamen, which are separated by a thin layer of white matter. Evidence from clinical lesion studies suggest that the neural circuits originating from the caudate nucleus and putamen may operate independently (Bansil et al., 2012; Bhatia & Marsden, 1994). Consistently, noninvasive functional parcellations of the basal ganglia show that the caudate and putamen are characterized by distinct patterns of resting state functional connectivity that are qualitatively reliable across individuals (Barnes et al., 2010). Furthermore, cortico‐striatal connectivity of large‐scale functional brain networks differs within the striatum, where the fronto‐parietal network (FPN) and default mode network (DMN) show preferential connectivity with the caudate, while the salience network shows preferential connectivity with the putamen (Karcher et al., 2019).

Multiple lines of evidence implicate the dorsal striatum in the pathophysiology of psychosis spectrum disorders (Cui et al., 2016; Dandash et al., 2014; Fornito et al., 2013; Fusar‐Poli & Meyer‐Lindenberg, 2013; Fusar‐Poli et al., 2011; Fusar‐Poli et al., 2010; Galindo et al., 2017; Hoffman et al., 2011; Howes et al., 2012; Karcher et al., 2019; Kirino et al., 2018; Koch et al., 2014; Laruelle, 2014; Li et al., 2018; Li et al., 2019; McCutcheon et al., 2019; Oh et al., 2020; Orliac et al., 2013; Peters et al., 2017; Quide et al., 2013; Salvador et al., 2010; Sarpal et al., 2017; Sarpal et al., 2015; Tu et al., 2013; Viher et al., 2019; Wang et al., 2015; Zhuo et al., 2014). A review by Laruelle (2014) and a meta‐analysis by Fusar‐Poli & Meyer‐Lindenberg (2013) show increased striatal dopamine levels in patients with schizophrenia. This finding has been replicated in medicated schizophrenia patients, antipsychotic drug‐naïve patients (Howes et al., 2012), high‐risk patients (Fusar‐Poli et al., 2011; Fusar‐Poli et al., 2010), and first‐degree relatives of patients with a psychotic disorder (Huttunen et al., 2008). It appears that this elevation is greatest in the caudate (Kegeles et al., 2010). Interestingly, striatal dysconnectivity, which may be a reflection of dopamine dysfunction, has been reported in subjects at high risk for psychosis, in first episode psychosis patients and chronic schizophrenia patients (Cui et al., 2016; Dandash et al., 2014; Fornito et al., 2013; Galindo et al., 2017; Hoffman et al., 2011; Karcher et al., 2019; Kirino et al., 2018; Koch et al., 2014; Li et al., 2018; Li et al., 2019; Oh et al., 2020; Orliac et al., 2013; Peters et al., 2017; Quide et al., 2013; Salvador et al., 2010; Sarpal et al., 2017; Sarpal et al., 2015; Tu et al., 2013; Viher et al., 2019; Wang et al., 2015; Zhuo et al., 2014), suggesting this may be a key pathophysiological finding. Given that both dopamine hyperactivity and functional dysconnectivity are primary focuses in the search for clinically relevant biomarkers of schizophrenia (Kraguljac et al., 2021), knowledge of striatal dysconnectivity as a key component of schizophrenia pathophysiology could help inform future diagnostic and/or treatment decisions.

Studies that investigated one or both of the striatal subregions separately show caudate dysconnectivity to the DMN (Dandash et al., 2014; Fornito et al., 2013; Galindo et al., 2017; Kirino et al., 2018; Salvador et al., 2010; Sarpal et al., 2015; Tu et al., 2013; Viher et al., 2019; Zhao et al., 2018), and dysconnectivity of the putamen to regions of the salience network (Dandash et al., 2014; Karcher et al., 2019; Koch et al., 2014; Li et al., 2019; Orliac et al., 2013; Peters et al., 2017; Sarpal et al., 2015). In a placebo‐controlled dopamine challenge study, a striking reduction in connectivity between the dorsal caudate and regions of the default mode network as well as an increase of putamen connectivity to the cerebellum were reported after L‐dopa administration (Kelly et al., 2009), underscoring that a dopaminergic modulation system may differentially affect connectivity of these striatal subregions.

Because all antipsychotic drugs work as dopamine D2 receptor antagonists, there has been a great deal of interest in understanding the relationship between striatal abnormalities in psychosis spectrum disorders and response to antipsychotic treatment. Short periods of antipsychotic treatment have been shown to ameliorate striatal dysfunction (Cadena et al., 2018; Chua et al., 2009; Sarpal et al., 2015) and provide evidence that dorsal striatal functional abnormalities in unmedicated patients with a schizophrenia spectrum disorder predicts response to antipsychotic treatment (Cadena et al., 2018; Kraguljac et al., 2016).

Our study had two goals. First, we attempted to determine patterns of dorsal striatal dysconnectivity in antipsychotic medication‐naïve or unmedicated patients with a psychosis spectrum disorder, separately for the caudate and putamen. Second, we aimed to determine if these patterns of dysconnectivity were predictive of subsequent response to antipsychotic treatment. Using resting state functional connectivity, which measures the temporal coherence of spontaneous neural activity between brain regions (Biswal et al., 1995; Damoiseaux et al., 2006), we evaluated functional connectivity of the caudate and putamen in two psychosis spectrum disorder cohorts: a cohort of medication‐naïve first episode psychosis patients and a cohort of unmedicated schizophrenia patients (along with matched healthy controls for each cohort). Based on the literature, we hypothesized that caudate dysconnectivity would show regional significance in areas of the DMN and that putamen dysconnectivity would show regional significance in areas associated the salience network. We further hypothesized that regions of significant dysconnectivity with either seed region would be predictive of response to antipsychotic treatment.

## 2 METHODS

### 2.1 Subjects

In this study, we present data from two independent psychosis spectrum disorder cohorts. As in our previous studies (Briend et al., 2020; Cadena et al., 2018; Kraguljac et al., 2016; Kraguljac et al., 2019; Kraguljac et al., 2016; Maximo et al., 2020; Nelson et al., 2020; Nelson et al., 2020; Nelson et al., 2018), patients were recruited from the emergency room, inpatient units, and outpatient clinics at the University of Alabama at Birmingham (UAB). Cohort 1 was comprised of 75 antipsychotic drug‐naïve first episode psychosis patients (FEP) with five or less lifetime days of antipsychotic medication exposure. Cohort 2 was comprised of 46 unmedicated patients with schizophrenia (SZ). Cohort 2 patients had been off of antipsychotic medications for at least 2 weeks at the time of enrollment. Approval for these studies was given by the UAB Institutional Review Board. Prior to enrollment, once it was determined a subject had the capacity to provide consent, written informed consent was acquired (Carpenter et al., 2000).

Both cohorts were enrolled in a 6‐week trial of risperidone using a flexible dosing regimen. In cohort 1 Risperidone was started at 0.5–1 mg and titrated in 1–2 mg increments; dosing was based on therapeutic and side effects. Two patients were switched to aripiprazole due to excessive side effect burden. Patients switched to aripiprazole started at 2–5 mg and titrated in 2.5–10 mg increments. Average risperidone dosage for cohort 1 was 4.4 mg (standard deviation 2.2 mg) and average aripiprazole dosage was 8.5 (standard deviation 9.2 mg). In cohort 2, risperidone was started at 1–3 mg and titrated in 1–2 mg increments. Average risperidone dosage for cohort 2 was 3.9 mg (standard deviation 1.67 mg). One subject from cohort 2 did not have medication data. For both cohorts, pill counts were done to monitor compliance. The use of concomitant psychotropic medications were permitted as clinically indicated. The number of patients taking concomitant medications in cohort 1 included: amphetamine salts (1), benztropine (23), diphenhydramine (1), hydroxyzine (1), lithium (1), lorazepam (4), sodium valproate (1), SSRIs (15), and trazodone (6), while concomitant medications for cohort 2 included: amitriptyline (1), benztropine (19), clonazepam (1), desvenlafaxine (1), divalproex sodium (1), mirtazapine (2), SSRIs (5) and trazodone (1). One patient from cohort 1 and three from cohort 2 did not have medication information. Cohorts 1 and 2 had seven and five treatment dropout patients by week 6, respectively.

Exclusion criteria included major neurological and/or medical conditions, a history of head trauma with loss of consciousness, substance use disorders (excluding nicotine [and cannabis in cohort 1 only]) inside 1 month of imaging, pregnancy or breastfeeding, or MRI contraindications. Two board‐certified psychiatrists (ACL and NVK) determined patient diagnoses through medical record review and consensus. Symptom severity was assessed using the Brief Psychiatric Rating Scale (BPRS) (Overall & Gorham, 1962). The Repeatable Battery for the Assessment of Neuropsychological Status (RBANS) was utilized to determine level of cognitive functioning (Randolph et al., 1998).

Healthy controls (HC) were also recruited for each cohort (63 in cohort 1; 41 in cohort 2) and group level matched for age, gender, and parental socioeconomic status (SES). In addition to the previously mentioned exclusion criteria, HC with personal or family (first‐degree relative) history of psychiatric illness were also excluded.

### 2.2 Data acquisition parameters

The data acquisition parameters described below were identical to those in our previous study (Nelson et al., 2020).

Dataset 1. Participants were scanned on a whole‐body 3T Siemens MAGNETOM Prisma MRI scanner using a 20 channel head coil.

Anatomical scans were acquired via a T1‐weighted MPRAGE (TR/TE = 2400/2.22 ms, flip angle 8°, 0.8 mm isotropic voxels).

Two resting state scans were acquired in opposing phase encoding directions (A > P and P > A; TR/TE = 1550/37.80 ms, flip angle = 71°, 2 mm isotropic voxels, 72 axial slices, 225 acquisitions in each direction). During the scan, subjects were instructed to keep their eyes open and stare passively ahead.

Dataset 2. Participants were scanned on a head‐only 3T Siemens MAGNETOM Allegra MRI scanner with a circularly polarized transmit/receive head coil.

Anatomical scans were acquired via a T1‐weighted MPRAGE (TR/TE = 2300/3.93 ms, flip angle = 12°, 1 mm isotropic voxels).

Resting state scans were acquired with a 5‐min gradient recalled echo‐planar imaging sequence (TR/TE = 2000/30 ms, flip angle = 70°, 6‐mm slice thickness, 1 mm gap, 30 axial slices, 225 acquisitions). During the scan, subjects were instructed to keep their eyes open and stare passively ahead.

### 2.3 Data preprocessing

To allow for signal equilibration, the first 10 frames were removed for each resting state run using FSL's topup (Glasser et al., 2013). Because resting state data was acquired in opposite phase encoding directions for cohort 1, these scans were merged and corrected for field inhomogeneity. Both data sets were preprocessed using the CONN toolbox version 18a (Whitfield‐Gabrieli & Nieto‐Castanon, 2012), as described in our previous study (Nelson et al., 2020).

The first BOLD time series eigenvariate from AAL atlas defined bilateral caudate and putamen seed regions were extracted and correlated to the time series of all other voxels creating individual caudate and putamen seed‐to‐voxel correlation maps for each subject (unit measurements were Pearson's r correlations). These correlation maps were subsequently converted into normally distributed values using Fisher's r‐to‐z transform.

### 2.4 Statistical analysis

Subject specific group‐level functional connectivity was obtained via one‐sample t‐tests. To test differences in functional connectivity between groups for each cohort (cohort 1: FEP vs matched HC; cohort 2: SZ vs matched HC), we binarized and unionized group‐level functional connectivity maps (for caudate and putamen seeds separately), then intersected each union mask with a gray matter mask. Group analyses were then performed using small volume correction (*p* < .01), and cluster corrected using threshold‐free cluster enhancement (TFCE) within each mask (Smith & Nichols, 2009). Covariates of no interest included framewise displacement (FD), age, and sex.

In order to evaluate the relationship between baseline resting state connectivity and subsequent treatment response (calculated as the % change from (A) baseline BPRS positive score to (B) week 6 score: (((B‐A)/A)×(−100) in each cohort. Group difference results (for caudate and putamen seeds separately) were used to create binarized masks. Regression analyses of treatment response on connectivity were then performed as above using the same small volume correction parameters, TFCE cluster correction, and the same covariates of no interest listed above.

In cohort 1, of the 75 FEP and 63 HC that completed resting state scans, four subjects (4 FEP and 0 HC) were excluded for excessive FD. Another eight FEP subjects lacked BPRS positive scores at baseline and/or week 6 and could not be included in treatment response analyses. In cohort 2, of the 46 SZ and 41 HC that completed the resting state scans, four subjects (4 SZ and 0 HC) were excluded for excessive FD. Another three SZ subjects lacked BPRS positive scores at baseline and/or week 6 and could not be included in treatment response analyses. Overall, final group comparison analyses comprised of 71 FEP and 63 HC in cohort 1 as well as 42 SZ and 41 HC in cohort 2. Final treatment response analyses included 64 FEP in cohort 1 and 39 SZ in cohort 2.

## 3 RESULTS

### 3.1 Demographics and clinical data

Neither cohort showed significant group differences for gender, age, or parental SES. Groups differed for movement with HC having a greater % of volumes retained in both cohorts and FEP having greater FD in cohort 1. To mitigate the impact of movement difference between groups, we used FD as a covariate of no interest. Patients showed marked clinical improvement based on change in baseline BPRS positive score after 6 weeks of treatment in both cohorts (from 11.35 at baseline to 5.38 in cohort 1 and from 9.74 at baseline to 5.38 in cohort 2). To better quantify the clinical characteristics of the patients, we compared RBANS scores to that of HC in both cohorts. For both cohorts, HC RBANS scores (including all subscales) were consistently higher (as expected) than patients. Table 1 shows results for all clinical and demographic measures.

**TABLE 1:** Demographics, clinical measures, and covariates^a^

Data set 1				
	FEP (*n* = 71)	HC (*n* = 63)	*t*/χ^2^	*p*
Gender (%male)	63.4	63.5	0.000	.989
Age	23.73 (6.00)	24.25 (5.91)	0.506	.614
Socioeconomic status^b^	5.46 (4.69)^c^	4.24 (4.02)	19.997	.220
Smoking (packs per day)	0.23 (0.40)^d^	0.20 (0.08)	−4.349	< .001
Baseline BPRS				
Total	49.62 (11.80)			
Positive	11.35 (3.37)			
Negative	5.79 (3.17)			
Week 6 BPRS^e^				
Total	32.61 (8.79)			
Positive	5.38 (3.05)			
Negative	5.69 (2.58)			
RBANS^f^				
Total	74.86 (14.97)	92.67 (10.95)	7.520	< .001
Immediate memory	81.88 (17.56)	101.33 (16.00)	6.344	< .001
Visuospatial/constructional	75.63 (17.13)	82.51(13.06)	2.462	.015
Language	84.19 (16.20)	97.39 (15.01)	4.630	< .001
Attention	81.42 (16.01)	101.75 (16.04)	6.967	< .001
Delayed memory	77.91 (14.56)	91.23 (8.66)	6.192	< .001
Resting state fMRI				
% of volumes retained after scrubbing	94.28 (6.51)	97.17 (4.07)	3.110	.002
Framewise displacement (mm)	0.31 (0.18)	0.23 (0.10)	−3.231	.002

Data set 2				
	SZ (*n* = 42)	HC (*n* = 41)	***t*/χ^2^**	***p***
Gender (%male)	71.4	75.6	.186	.666
Age	27.64 (9.61)	28.73 (9.37)	.522	.603
Socioeconomic status^b^	6.62 (5.81)^c^	5.27 (4.23)	15.790	.261
Smoking (packs per day)	0.32 (0.48)	0.26 (0.47)	‐0.534	.595
APD naïve (yes/ no)	27/15			
Baseline BPRS				
Total	49.67 (9.18)			
Positive	9.74 (3.49)			
Negative	7.52 (3.30)			
Week 6 BPRS^e^				
Total	32.59 (9.70)			
Positive	5.38 (2.49)			
Negative	5.77 (2.61)			
RBANS^f^				
Total	70.78 (14.81)	91.02 (12.71)	6.608	< .001
Immediate memory	77.90 (17.06)	97.39 (14.33)	5.573	< .001
Visuospatial/constructional	70.28 (15.81)	84.37 (17.73)	3.772	< .001
Language	83.65 (11.67)	98.78 (15.10)	5.038	< .001
Attention	77.10 (21.91)	95.80 (18.51)	4.155	< .001
Delayed memory	73.83 (20.22)	92.71 (9.30)	5.378	< .001
Resting state fMRI				
% of volumes retained after scrubbing	93.11 (7.64)	96.96 (3.81)	−2.919	.005
Framewise displacement (mm)	0.36 (.19)	0.29 (0.14)	−1.949	.055

Abbreviations: APD, antipsychotic drug; BPRS, Brief Psychiatric Rating Scale (positive subscale included conceptual disorganization, hallucinatory behavior, and unusual thought content; FEP, first episode psychosis patients; fMRI, functional magnetic resonance imaging.; HC, healthy controls; negative subscale included emotional withdrawal, motor retardation, and blunted affect); RBANS, Repeatable Battery for the Assessment of Neuropsychological Status; SZ, patients with schizophrenia.

^a^ Mean (standard deviation) unless indicated otherwise.

^b^ Parental socioeconomic ranks determined from Diagnostic Interview for Genetic Studies (1–18 scale); higher rank (lower numerical value) corresponds to higher socioeconomic status.

^c^ Data Set 1: Data not available for six FEP subjects; *n* = 65; Data Set 2: Data not available for three SZ subjects; n = 39.

^d^ Data Set 1: Data not available for one FEP subject; FEP, *n* = 70.

^e^ Data Set 1: Data not available for eight FEP subjects; FEP *n* = 64; Data Set 2: Data not available for three SZ subject; SZ, *n* = 39.

^f^ Data Set 1: Data not available for seven FEP subjects or six HC; FEP, *n *= 64, HC, *n* = 57; Data Set 2: Data not available for two SZ subjects; SZ *n* = 40.

### 3.2 Caudate resting state functional connectivity

**FIGURE 1:**
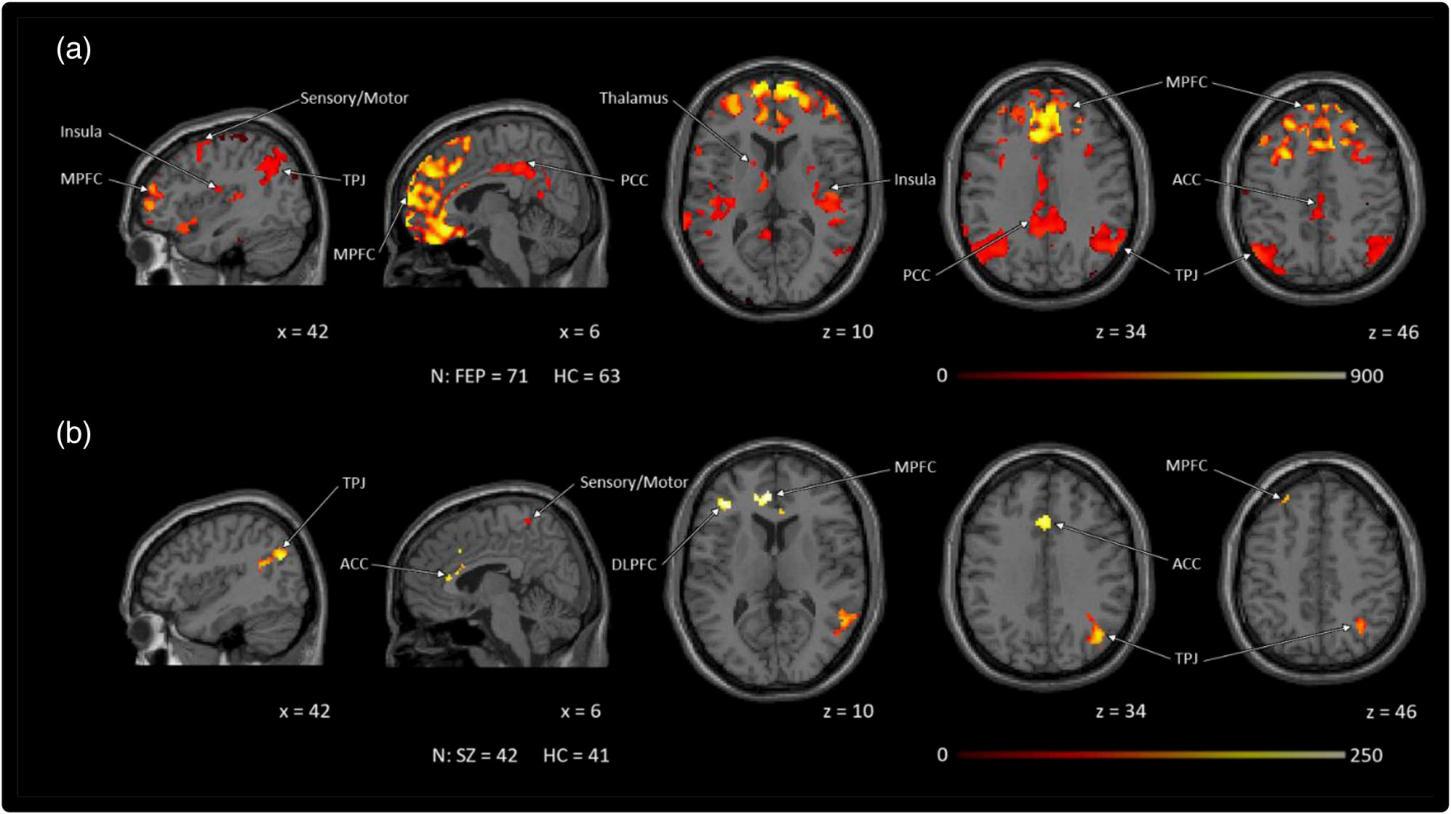
Caudate resting state functional connectivity: patient versus control group difference results for both (a) FEP and (b) SZ, showed hypoconnectivity between the bilateral caudate and several brain regions, particularly the default mode network. Though the results were much more robust in cohort 1, both cohorts had hypoconnectivity to the MPFC, PCC, TPJ, ACC, and sensory/motor cortex. FEP also showed hypoconnectivity to the insula and thalamus. All group difference analyses were masked and performed using small volume correction (*p* < .01), and then cluster corrected using threshold‐free cluster enhancement within each mask. Age, sex, and framewise displacement were treated as covariates. Abbreviations: ACC, anterior cingulate cortex; DLPFC, dorsolateral prefrontal cortex; FEP, antipsychotic drug‐naïve first episode psychosis patients; HC, healthy controls; MPFC, medial prefrontal cortex; PCC, posterior cingulate cortex; TPJ, temporoparietal junction; SZ, unmedicated patients with schizophrenia.

In both cohorts, patients showed hypoconnectivity between the bilateral caudate and regions of the DMN: the medial prefrontal cortex (MPFC), posterior cingulate cortex (PCC; in FEP only), temporoparietal junction (TPJ; bilaterally in FEP and the left TPJ in unmedicated SZ). Both cohorts also showed hypoconnectivity to anterior cingulate cortex (ACC), sensory/motor areas and the temporal gyri. In cohort 1, FEP also had hypoconnectivity to the bilateral insula, fusiform gyrus, left putamen and caudate, and the right thalamus and amygdala (Figure 1).

### 3.3 Caudate dysconnectivity and treatment response

**FIGURE 2:**
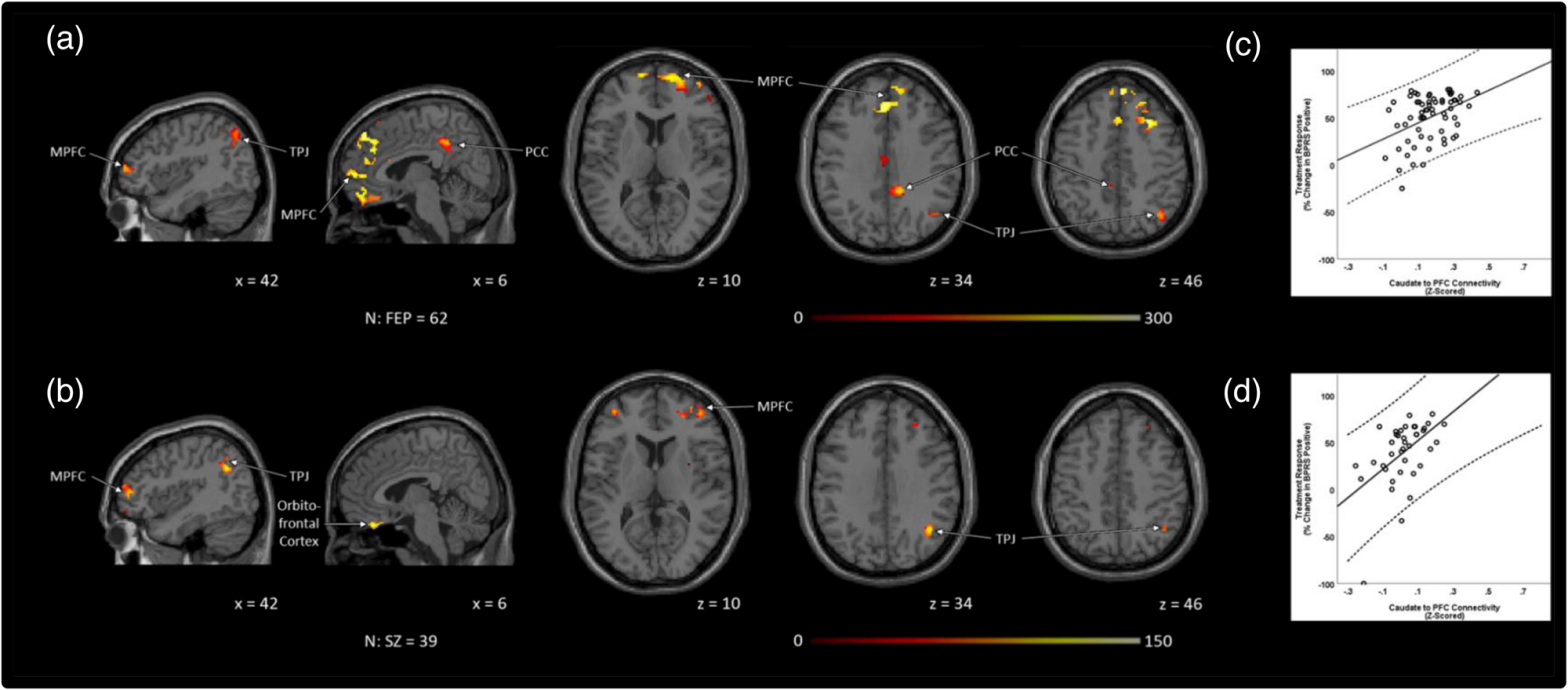
Caudate dysconnectivity predicts treatment response. (a) Among FEP and (b) SZ, caudate dysconnectivity to the MPFC, TPJ, and PCC in cohort 1 (all areas of the DMN) were predicative of better treatment response. All treatment response analyses were masked and performed using small volume correction (*p* < .01), and then cluster corrected using threshold‐free cluster enhancement within each mask. Age, sex, and FD were treated as covariates. Treatment response was based on % change in BPRS‐positive score from baseline to week 6. Average z‐scored connectivity beta weights were extracted from PFC clusters in (c) cohort 1 and (d) cohort 2 and plotted with treatment response. Abbreviations: BPRS, The Brief Psychiatric Rating Scale; FEP, antipsychotic drug‐naïve first episode psychosis patients; HC, healthy controls; MPFC, medial prefrontal cortex; PCC, posterior cingulate cortex; TPJ, temporoparietal junction; SZ, unmedicated patients with schizophrenia.

Among FEP, dysconnectivity between the caudate and the MPFC, right insula, PCC, ACC, and left TPJ was predictive of better subsequent treatment response. Among unmedicated SZ, dysconnectivity between the caudate and the MPFC, right insula, left TPJ, and the sensory/motor area was also predictive of better treatment response. To determine the relationship between caudate functional connectivity and response to treatment we extracted averaged z‐scored connectivity beta weights from PFC clusters predictive of treatment response in both patient cohorts. Plots consistently showed that in both cohorts, greater resting state connectivity between the PFC and caudate was associated with better response to treatment (Figure 2).

### 3.4 Putamen resting state functional connectivity

**FIGURE 3:**
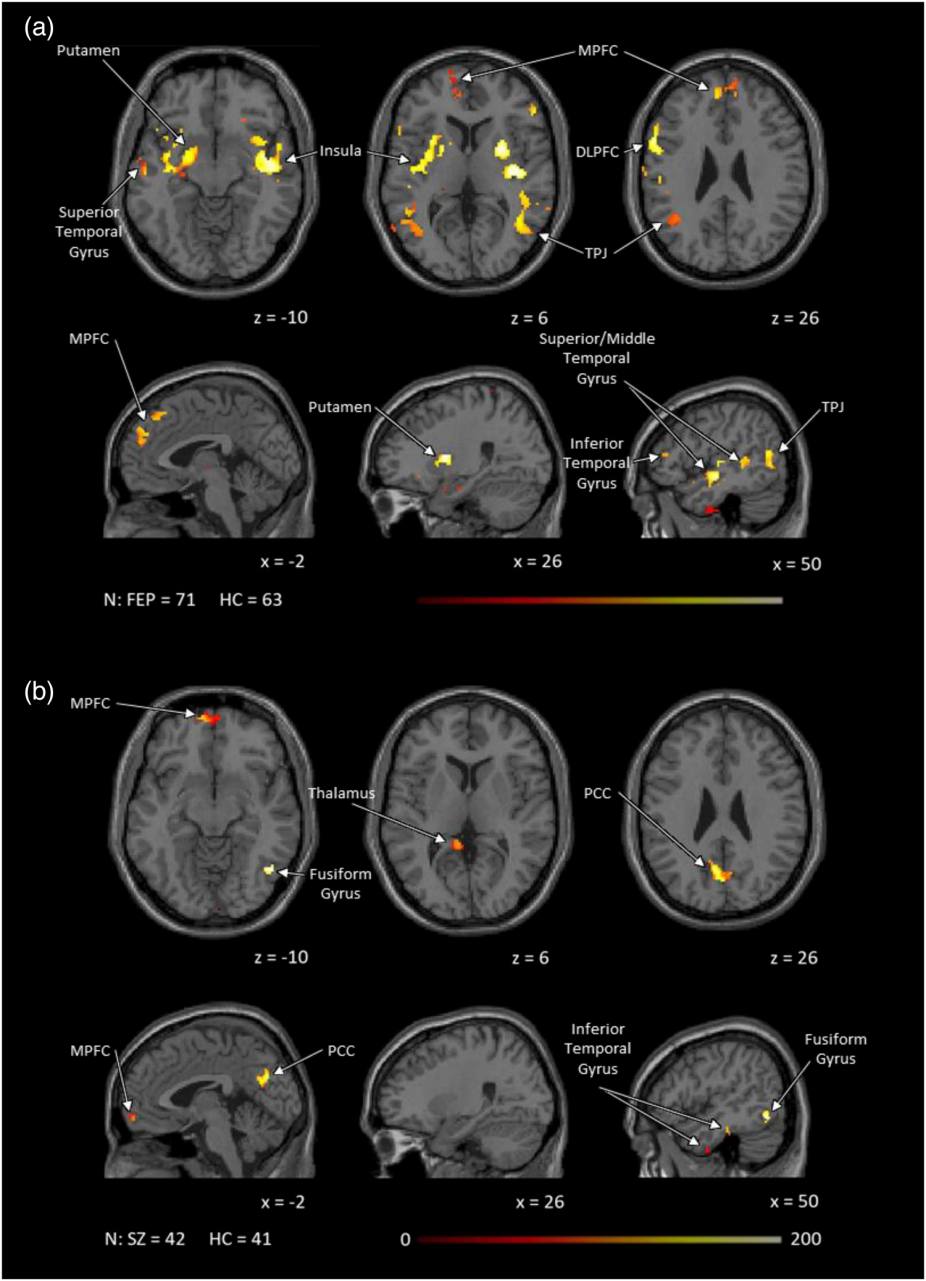
Putamen resting state functional connectivity: patient versus control group difference results for both (a) FEP and (b) SZ, showed hypoconnectivity between the bilateral putamen and the DMN including the MPFC in both cohorts the TPJ among FEP and the PCC among SZ. FEP also showed hypoconnectivity to the insula, temporal gyri, and right DLPFC. All group difference analyses were masked and performed using small volume correction (*p* < .01), and then cluster corrected using threshold‐free cluster enhancement within each mask. Age, sex, and framewise displacement were treated as covariates. Abbreviations: DLPFC, dorsolateral prefrontal cortex; FEP, antipsychotic drug‐naïve first episode psychosis patients; HC, healthy controls; MPFC, medial prefrontal cortex; PCC, posterior cingulate cortex; TPJ, temporoparietal junction; SZ, unmedicated patients with schizophrenia.

In both cohorts, results showed hypoconnectivity of the putamen to the MPFC. FEP also had hypoconnectivity bilaterally to the hippocampus, insula, sensory/motor cortex, fusiform and temporal gyri, and TPJ, as well as the right dorsolateral prefrontal cortex (DLPFC) and dorsal ACC. Unmedicated SZ showed hypoconnectivity to the left parahippocampus, right anterior precuneus and retrosplenial cortex, and PCC (Figure 3).

### 3.5 Putamen dysconnectivity and treatment response

**FIGURE 4:**
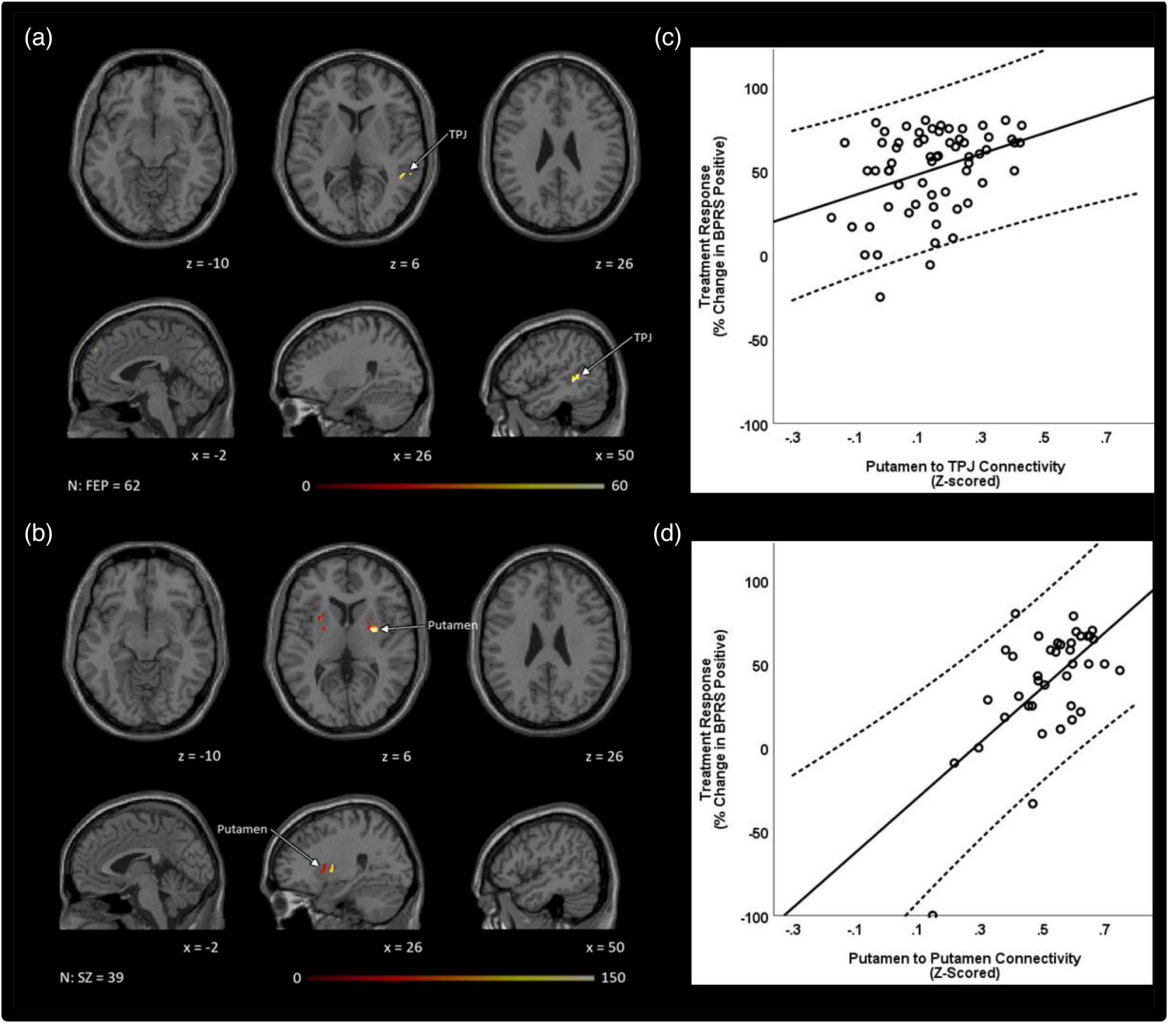
Putamen dysconnectivity predicts treatment response. (a) In FEP, putamen dysconnectivity to the left TPJ was predictive of better response to treatment. (b) In unmedicated SZ within putamen dysconnectivity was predicative of better treatment response. All treatment response analyses were masked and performed using small volume correction (*p *< .01), and then cluster corrected using threshold‐free cluster enhancement within each mask. Age, sex, and FD were treated as covariates. Treatment response was based on % change in BPRS‐positive score from baseline to week 6. Averaged z‐scored connectivity beta weights were extracted from (c) the TPJ in cohort 1 and (d) the left putamen in cohort 2 and plotted with treatment response. Abbreviations: BPRS, The Brief Psychiatric Rating Scale; FEP, antipsychotic drug‐naïve first episode psychosis patients; HC, healthy controls; TPJ, temporoparietal junction; SZ, unmedicated patients with schizophrenia.

In cohort 1, dysconnectivity between the left TPJ and putamen was predictive of treatment response. In cohort 2, only dysconnectivity within the putamen bilaterally was predictive of treatment response. To determine the relationship between putamen connectivity and treatment response, we extracted averaged z‐scored connectivity beta weights from clusters predictive of treatment response in patients in both cohorts (the TPJ in cohort 1 and the left putamen in cohort 2). Plots in both cohorts showed that greater putamen connectivity to each cluster was associated with better response to treatment (Figure 4).

### 3.6 Qualitative comparisons of findings between cohorts

Overall, caudate hypoconnectivity patterns showed a great deal of overlap across patient cohorts including patterns where hypoconnectivity predicted treatment response, although these patterns were much more robust in cohort 1 than in cohort 2. Patterns of putamen hypoconnectivity also showed some degree of overlap across patient cohorts, but the caudate showed more widespread hypoconnectivity (particularly to the DMN) and treatment response prediction than the putamen in both cohorts. Furthermore, while both cohorts showed hypoconnectivity to regions of the DMN, FEP showed strongest putamen dysconnectivity with the insula.

## 4 DISCUSSION

To our knowledge, this is the first study to assess resting state functional connectivity of two anatomically distinct subregions of the dorsal striatum in two cohorts of patients with a psychosis spectrum disorder. Our results demonstrate the presence of dysconnectivity between the dorsal striatum and large‐scale brain networks in psychosis spectrum patients, where reduced connectivity appears most prominent between the caudate and the DMN as well as the putamen and areas of the salience network (most robustly seen in FEP). Importantly, baseline dysconnectivity of both striatal subregions was predictive of subsequent antipsychotic treatment response, where greater baseline connectivity abnormalities were associated with poorer outcomes. This data adds to the existing literature implicating cortico‐striatal dysconnectivity in the psychosis pathology and supports that dysconnectivity of these regions, likely secondary to dopamine dysfunction, is clinically relevant.

Our findings of differential dysconnectivity of the two striatal subregions to areas of large‐scale cortical networks is largely in agreement with several other studies. These previous studies report caudate hyperconnectivity to the DMN (Kirino et al., 2018; Salvador et al., 2010) as well as putamen hypoconnectivity to the salience network (Karcher et al., 2019; Orliac et al., 2013) and the insula specifically (Koch et al., 2014; Peters et al., 2017) in chronic schizophrenia patients, though we report hypo‐ rather than hyperconnectivity between the caudate and DMN. In FEP, Fornito et al. (2013) reported a dorsal to ventral gradient of hypo‐ to hyperconnectivity between the caudate and prefrontal regions. In this study, a gradient was also noted for frontostriatal connectivity of the putamen, suggesting a complex pattern of dysconnectivity in the early illness stages. Others have reported caudate hyperconnectivity to the primary motor cortex (Oh et al., 2020) and the prefrontal cortex (Sarpal et al., 2015), and putamen hypo‐ or hyperconnectivity to the insula (Dandash et al., 2014; Li et al., 2019; Sarpal et al., 2015) and ACC (Oh et al., 2020) in FEP. Interestingly, in patients reporting hallucinations, increased putamen to prefrontal connectivity was also associated with greater positive symptom severity (Cui et al., 2016). Discrepancies between reports in FEP may be due to differences in methodology or sample characteristics such as the inclusion of medicated subjects at the time of scanning (Fornito et al., 2013; Oh et al., 2020), or analyzing caudate and putamen subfields ipsilaterally instead of using each whole structure bilaterally as seed regions (Sarpal et al., 2015).

It is possible that the dysconnectivity between the dorsal striatum and large‐scale brain networks is related to dopamine dysfunction, as both medicated and drug‐naïve patients show increased striatal dopamine (Fusar‐Poli & Meyer‐Lindenberg, 2013; Howes et al., 2012; Kegeles et al., 2010; Laruelle, 2014). Furthermore, antipsychotic medications, which modulate dopaminergic neurotransmission, ameliorate dorsal striatal abnormalities. For example, antipsychotic‐naïve FEP showed increases in both caudate to prefrontal cortex and putamen to insula connectivity after 12 weeks of treatment (Sarpal et al., 2015). In earlier work, we have shown that decreased caudate task activation prior to antipsychotic treatment was predictive of better treatment response and an increase in putamen activity from baseline to after 6 weeks of treatment was associated with a favorable response to antipsychotic medication (Cadena et al., 2018). We have also shown that greater baseline resting state connectivity between the caudate and hippocampus in unmedicated schizophrenia patients was predictive of better subsequent treatment response (Kraguljac et al., 2016), which is similar to findings by Sarpal et al. (2016), who found that striatal connectivity to the insula, cingulate cortex, and PFC was predictive of treatment response in FEP. Interestingly, their results also showed an anterior–posterior gradient of striatal connectivity where increased connectivity to posterior brain regions and decreased connectivity to anterior brain regions were predictive of better response.

Our results suggest that two distinct dysconnectivity patterns of the dorsal striatum may be relevant for antipsychotic treatment response, where caudate dysconnectivity to areas of the DMN was predictive of treatment response in both cohorts, but spatial patterns of dysconnectivity predictive of treatment response were not consistent between cohorts for the putamen seed. It is tempting to speculate that the caudate dysconnectivity to the DMN may reflect an underlying dopamine dysfunction in psychosis spectrum disorders that can be ameliorated with antipsychotic medication treatment.

### 4.1 Strengths and limitations

One of the seminal strengths of this study was the evaluation of coequal biological measures obtained among two independent cohorts on disparate scanners with disparate acquisition protocols. The fact that both cohorts had no antipsychotic medication exposure at the time of the scan is another important factor. Additionally, illness chronicity and prior antipsychotic medication exposure confounds were fully mitigated in cohort 1 with the exclusive inclusion of FEP, and to a lesser degree in cohort 2 where the dataset consisted of approximately two‐thirds of antipsychotic‐naïve patients. Moreover, similar data preprocessing and identical quality control parameters were used for both resting state datasets in order to minimize variance across data sets. Some of this study's limitations should be noted though. First, our interpretation result replicability was based on face validity. We did not perform formal tests of replicability across cohorts, as it was outside the scope of this study. Second, though evidence showing the relationship between increased motor abnormalities and poor functional outcome demonstrates the clinical relevance that motor abnormalities play psychosis spectrum disorders (Pieters et al., 2021), we did not systematically assess motoric side effects in this study. However, we did motion scrub functional scans at the subject level and included FD as a covariate of no interest to help mitigate any potential movement confounds that may have existed within our functional connectivity data. Third, the superior signal‐to‐noise ratio inherent to the more advanced acquisition parameters used for cohort 1 detected abnormal resting state connectivity patterns that may have been below the detectable threshold in cohort 2.

## 5 CONCLUSIONS

In two patient cohorts, we observed two distinct patterns of hypoconnectivity for the caudate and putamen, while the patterns of dysconnectivity to each separate seed region were similar across cohorts. Furthermore, we showed that spatial patterns of caudate dysconnectivity to the DMN that predicted treatment response were also similar across cohorts. The replicability of these findings, including in a cohort of medication‐naïve FEP, helps establish both putamen and particularly caudate dysconnectivity as useful biological markers of psychosis and predictors of response to antipsychotic medication.

## CONFLICT OF INTEREST

Kraguljac serves as consultant for Neurocrine Biosciences, Inc. All other authors report no relevant biomedical financial interests or potential conflicts of interest.

### PEER REVIEW

The peer review history for this article is available at https://publons.com/publon/10.1002/brb3.2625.

## Data Availability

MRI data might be obtained upon request by contacting the corresponding author.
